# Quality Evaluation of Decoction Pieces of Gardeniae Fructus Based on Qualitative Analysis of the HPLC Fingerprint and Triple-Q-TOF-MS/MS Combined with Quantitative Analysis of 12 Representative Components

**DOI:** 10.1155/2022/2219932

**Published:** 2022-02-26

**Authors:** Jing Xu, Rongrong Zhou, Lin Luo, Ying Dai, Yaru Feng, Zhihua Dou

**Affiliations:** ^1^School of Pharmacy, Nantong University, Nantong 226019, Jiangsu, China; ^2^Nantong Third People's Hospital, Nantong University, Nantong 226006, Jiangsu, China; ^3^School of Pharmacy, Nanjing University of Chinese Medicine, Nanjing 210023, Jiangsu, China

## Abstract

In this study, quality evaluation (QE) of 40 batches of decoction pieces of Gardeniae Fructus (GF) produced by different manufacturers of herbal pieces was performed by qualitative analysis of the HPLC fingerprint and ultra-fast liquid chromatography (UFLC)-triple-Q-TOF-MS/MS combined with quantitative analysis of multiple components, which we established previously for QE of traditional medicine. First, HPLC fingerprints of 40 samples were determined, and the common peaks in the reference fingerprint were assigned. Second, the components of the common peaks in the HPLC fingerprints were identified by UFLC-triple-Q-TOF-MS/MS. Finally, the contents of the components confirmed by reference substances were measured. The results showed that there were 28 common peaks in the HPLC fingerprints of 40 samples. The components of these 28 common peaks were identified as 13 iridoids, 4 crocins, 7 monocyclic monoterpenoids, 3 organic acids, and 1 flavonoid. Of these, a total of 12 components, including 7 iridoids of geniposide, shanzhiside, geniposidic acid, deacetyl asperulosidic acid methyl ester, gardenoside, scandoside methyl ester, and genipin gentiobioside, 2 crocins such as crocin I and crocin II, 1 monocyclic monoterpenoid of jasminoside B, 1 organic acid of chlorogenic acid, and 1 flavonoid of rutin, were unambiguously identified by comparison with reference substances. There were certain differences in the contents of these 12 components among 40 samples. The geniposide content ranged from 37.917 to 72.216 mg/g, and the total content of the 7 iridoids ranged from 59.931 to 94.314 mg/g.

## 1. Introduction

Gardeniae Fructus (GF), the desiccative ripe fruit of *Gardenia jasminoides* Ellis (Rubiaceae), is a well-known and frequently used traditional medicine officially recorded in the Chinese and Japanese Pharmacopoeias [1–3]. To date, nearly 200 phytochemicals have been isolated and identified from GF [1, 4–9], which mainly include iridoids, crocins, monocyclic monoterpenoids, organic acids, and flavonoids [1, 10]. The representative components of iridoids include geniposide, genipin gentiobioside, gardenoside, shanzhiside, deacetyl asperulosidic acid methyl ester (DAAEM), and scandoside methyl ester (SME) [11, 12], crocins include crocin I and crocin II [13], monocyclic monoterpenoids include jasminoside A, jasminoside B, and 6′-O-trans-sinapoyl jasminoside A [14], and organic acids and flavonoids including chlorogenic acid and rutin [15].

GF and its components exhibit a broad range of pharmacological activities, such as hepatoprotective and anti-inflammatory [16, 17], renoprotective [8], antidiabetic and antioxidant [18, 19], antidepressant [20], antiviral [21], antithrombotic [22], and neuroprotective activities [23]. However, recent research results also show that high doses of GF and iridoids have certain hepatotoxicity and nephrotoxicity [24–26]. In other words, GF and iridoids have both toxic and protective effects on the liver and kidney. Therefore, strictly controlling the quality of GF is very important to ensure the safety and effectiveness of clinical medications.

**Figure 1:**
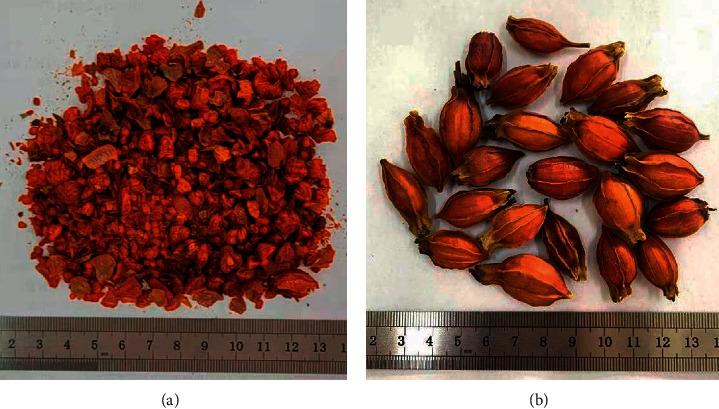
Decoction pieces of GF (a) and original medicinal materials of GF (b).

The original medicinal materials of GF can only be used in the clinic after being processed into decoction pieces [27]. Decoction pieces of GF (Figure 1(a)) are the product of original medicinal materials of GF after removing impurities and crushing (Figure 1(b)) [2]. The quality of GF decoction pieces is directly related to the safety and effectiveness of clinical medication. At present, there are some literature reports on the quality evaluation (QE) of original medicinal materials of GF [28–32], but there is no report on the QE of GF decoction pieces. Therefore, in this study, QE of 40 batches of GF decoction pieces produced by different manufacturers of herbal pieces was performed by qualitative analysis of the HPLC fingerprint and ultra-fast liquid chromatography (UFLC)-triple-Q-TOF-MS/MS combined with quantitative analysis of multiple components, which we established previously for QE of traditional medicine [33].

## 2. Experimental

### 2.1. Chemicals and Reagents

Reference substances geniposide (no. 110749–201718 with a purity of ≥97.6% (HPLC)), DAAME (no. 111786–201602 with a purity of ≥94.3%), crocin I (no. 111588–201202 with a purity of ≥91.1%), and crocin II (no. 111589–201103 with a purity of ≥91.9%) were purchased from the National Institutes for Food and Drug Control (Beijing, China). Shanzhiside (no. CHB161228), geniposidic acid (no. CHB161101), gardenoside (no. CHB180124), SME (no. CHB160931), genipin gentiobioside (no. CHB160720), jasminoside B (no. CHB180326), chlorogenic acid (no. CHB170713), and rutin (no. CHB170303) were purchased from Chengdu Chroma Biotechnology Co., Ltd. (Chengdu, China) (all substances with a purity of ≥98%). HPLC-grade methanol and LC/MS-grade acetonitrile were purchased from Fisher Scientific (Fair Lawn, NJ, USA). HPLC-grade formic acid and purified water were purchased from Nanjing Chemical Reagent Co., Ltd. (Nanjing, China) and Wahaha Group Co., Ltd. (Hangzhou, China), respectively.

### 2.2. Samples and Sample Preparation

Forty batches of GF decoction pieces produced by different manufacturers of herbal pieces were purchased from different large TCM hospitals in China; the information on all 40 samples is given in Table 1.

GF decoction pieces were ground into powders before use. Powder samples (0.1 g) were weighed accurately and placed in a 50 mL brown volumetric flask; approximately, 49 mL of 50% (v/v) methanol was added; the mixture was then extracted by ultrasonication (200 W, 53 kHz) for 30 min. After cooling to room temperature, 50% (v/v) methanol was added for calibration of the volumetric flask and shaken well; the mixture was filtered through a 0.22 *μ*m filter membrane, and the filtrate was taken as a sample solution.

### 2.3. Preparation of Reference Substance Solutions

Twelve reference substance stock solutions with a concentration range of 0.1–4 mg/mL were prepared by accurately weighing appropriate amounts of 12 reference substances and dissolving them in 50% (v/v) methanol.

Appropriate amounts of each reference substance stock solution were precisely measured, mixed together, and diluted with 50% (v/v) methanol. Thus, the mixed reference substance solution for qualitative analysis in a concentration range of 1–67 *μ*g/mL of each compound was prepared.

Working solution A in a concentration range of 3–381 *μ*g/mL of each compound for quantitative analysis was prepared by the same method as that used in preparing the mixed reference substance solution for qualitative analysis. Working solutions B, C, and D were prepared by diluting working solution A with 50% methanol to 2, 5, and 10 times its initial volume, respectively.

### 2.4. Chromatographic Conditions for HPLC Fingerprint and Quantitative Analysis

Determination of the HPLC fingerprint and quantitative analysis of 12 components were performed on an HPLC system equipped with an e2695 separation unit, a 2998 PDA detector, and an Empower 3 data processing system (Waters Corp., Milford, MA, USA). Chromatographic separation was performed on a Symmetry C_18_ column (4.6 mm × 250 mm, 5 *μ*m, Waters Corp., USA). The column was maintained at 30°C. Acetonitrile (A) and 0.1% (v/v) formic acid (B) were used as mobile phases using the following gradient elution program: 0–5 min, 2% A; 5–10 min, 2–5% A; 10–45 min, 5–15% A; 45–80 min, 15–40% A; 80–82 min, 40–98% A. The injection volume of sample solution was 30 *μ*L at a flow rate of 1.0 mL/min. The wavelength for the determination of fingerprints and contents of the 7 iridoids, rutin, and jasminoside B was set at 254 nm, and those for the determination of contents of chlorogenic acid and the 2 crocins were set at 324 nm and 430 nm, respectively.

### 2.5. Validation of the HPLC Method for Fingerprint Analysis

By using peak **11** (genipin gentiobioside) as the reference peak and the relative standard deviation (RSD) value of the relative peak area (RPA) and the average relative retention time (RRT) of the 28 common peaks as measurement values, the HPLC method for fingerprint determination was validated with precision, stability, and repeatability tests. The precision was determined by six replicate injections of the same sample (S1) solution. The stability test was performed by injecting the sample solution (S1) at 0, 6, 12, 18, 24, and 36 h after preparation. The repeatability was evaluated by six sample solutions prepared in parallel from S1.

### 2.6. Establishment and Similarity Analysis of the HPLC Fingerprint

The chromatographic data of 40 samples were imported into the Similarity Evaluation System for Chromatographic Fingerprint of Traditional Chinese Medicine software (Version 2012, Chinese Pharmacopoeia Commission, Beijing, China). The reference chromatogram was established using the chromatogram of sample 1 as the reference, and common peaks in this reference chromatogram were assigned. The similarities between sample chromatograms and reference chromatogram were calculated using the abovementioned software.

### 2.7. Mass Spectrometry Conditions for UFLC-Triple-Q-TOF-MS/MS Analysis

Identification of the components of common peaks in the HPLC fingerprint was performed on a UFLC-triple-Q-TOF-MS/MS system. Component separation was performed on a UFLC system (equipped with an LC-20AD XR quaternary pump, an SIL-20AC XR autosampler, and an SPD-M20 A DAD detector, Shimadzu, Kyoto, Japan) by using the same column with the same mobile phases and gradient conditions as mentioned above. The injection volumes of both the mixed reference substance solution and sample solution were 20 *μ*L. After component separation by UFLC, a Triple TOF 4600 system (AB SCIEX, Framingham, USA) was employed to acquire mass spectra in the negative ion mode with a DuoSpray source. The mass spectrometric parameters were set as follows: curtain gas (CUR) 35 psi, nebulizer gas (gas (1)) 65 psi, heater gas (gas (2)) 65 psi, ion spray voltage 4500 V, and source temperature 550°C. The TOFMS-IDA-10MS/MS method was used to obtain mass spectrometry data, and relevant parameters were set as follows: collision energy (CE) −10 eV, decluster potential (DP) −80 V, accumulation time 250 ms, mass range for TOF-MS detection 115–2000 Da, CE −35 eV, collision energy spread (CES) 15 eV, DP −80 V, accumulation time 100 ms, and mass range for the TOF-MS/MS detection 50–2000 Da. LC–MS/MS data were analyzed using PeakView mass spectrometry analysis software (Version 1.6, AB SCIEX, USA).

### 2.8. Method Validation of the Quantitative Analysis

The quantitative analysis method was validated by investigating the linear relationship, limit of detection (LOD), limit of quantitation (LOQ), precision, stability, repeatability, and recovery test of 12 components. The linear relationship was investigated by precisely injecting working solution A (10, 20, 30, and 40 *μ*L) and working solutions B, C, and D (10 *μ*L of each solution) into the HPLC system to calculate the regression equation, correlation coefficient, and linear range for all 12 components. After diluted, working solution D was injected into the HPLC system many times; LOQ and LOD were determined on the basis of signal-to-noise ratios of 10 : 1 and 3 : 1, respectively. Intraday precision, interday precision, and stability were assessed by RSDs of the peak areas of the 12 components. The intraday precision was determined by six consecutive injections of 30 *μ*L working solution A, and the interday precision was determined by six replicate injections of 30 *μ*L working solution A, twice per day over 3 consecutive days. The stability test was carried out by using the peak areas of the 7 iridoids, rutin, and jasminoside B at 254 nm, chlorogenic acid at 324 nm, and 2 crocins at 430 nm detected in Section 2.5 of the stability test. By calculating the contents of 12 components according to the peak areas of the 7 iridoids, rutin, and jasminoside B at 254 nm, chlorogenic acid at 324 nm, and 2 crocins at 430 nm detected in Section 2.5 of the repeatability test, and using the values of RSDs, the repeatability test was examined. For the recovery test, approximately 0.05 g S1 powder was precisely weighed, and 12 reference substance stock solutions were added at a sample/reference substance ratio of 1 : 1. Six sample solutions prepared in parallel by this method were analyzed, and the average recovery and RSDs of 12 components were calculated.

## 3. Results and Discussion

### 3.1. Validation of the Method for HPLC Fingerprint Analysis

The RSDs of RPA and RRT for precision were no more than 4.56% and 0.14%, those for stability did not exceed 4.84% and 0.20%, and those for repeatability were less than 4.87% and 0.21%, respectively. The results met the fingerprinting quality standards for TCM injections [34].

### 3.2. Establishment and Similarity Analysis of the HPLC Fingerprint

**Figure 2:**
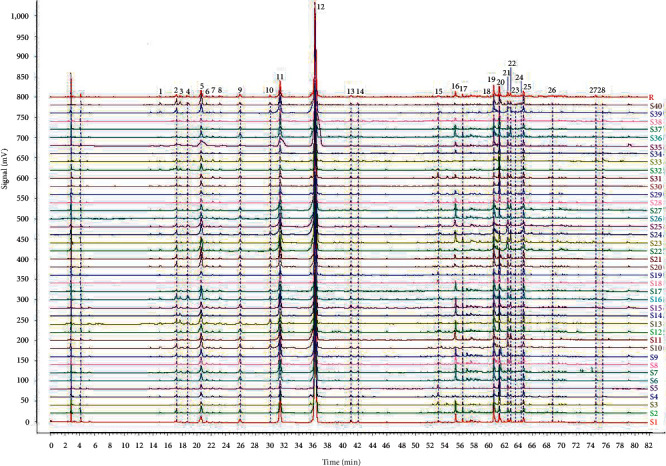
Chromatograms of 40 samples (S1–S40) and reference chromatogram (R).

As shown in Figure 2 and Table 1, 28 common peaks in the reference chromatogram were assigned. Similarities between the sample chromatograms and the reference chromatogram were all greater than 0.98.

### 3.3. Identification of the Common Peaks by Triple-Q-TOF-MS/MS

**Figure 3:**
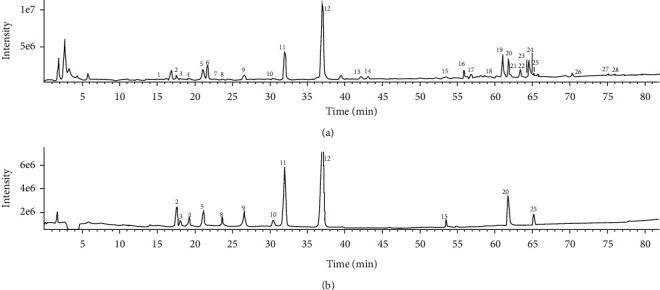
Total ion chromatograms of samples (a) and mixed reference substances (b).

**Figure 4:**
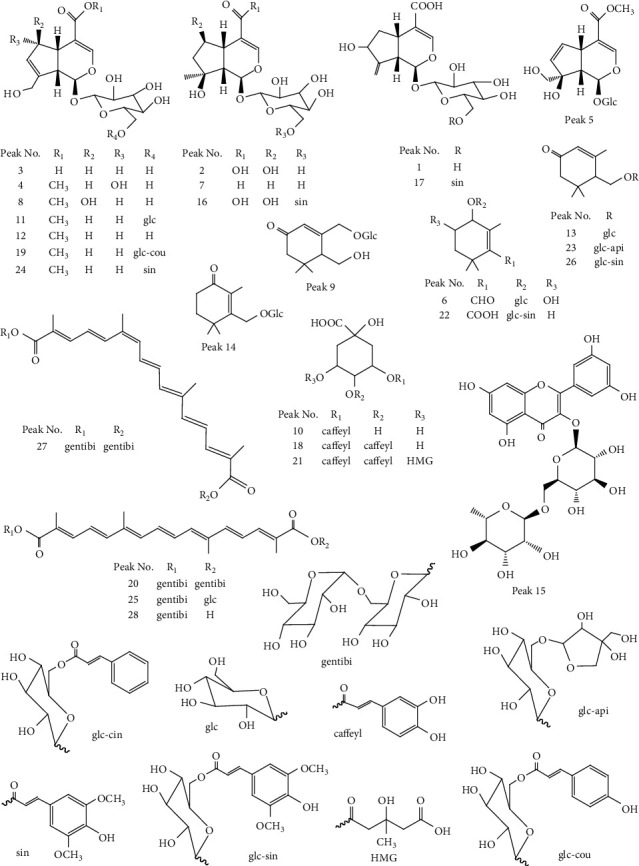
Structures or possible structures of the components of 28 common peaks.

A comparison between the negative ion mode and the positive ion mode revealed that the negative ion mode was much richer in information and thus was chosen for MS analysis. First, the total ion chromatograms of the sample and mixed reference substances (Figure 3) were extracted using PeakView software. Second, the mass spectral data and dissociative rules of the reference substances were summarized, and it was revealed that the quasimolecular ion [M-H]^−^ and/or [M+Cl]^−^ could be selected as the precursor ions to generate MS/MS product ion spectra. Finally, the retention time, quasimolecular ion, and MS/MS fragmentation patterns were compared between samples and reference substances or those reported in the literature. Online retrieval was performed in the database of PubChem (http://pubchem.ncbi.nlm.nih. Gov); therefore, the components of the 28 common peaks in the HPLC fingerprint were identified (the mass spectral data are given in Table 2, and the structures or possible structures of the components of 28 common peaks are shown in Figure 4).

As given in Table 2 and Figure 4, the 28 identified components include 13 iridoids, 4 crocins, 7 monocyclic monoterpenoids, 3 organic acids, and 1 flavonoid, of which, 12 components were unambiguously identified by comparison with the reference substances, including 7 iridoids shanzhiside (peak **2**), geniposidic acid (peak **3**), DAAME (peak **4**), gardenoside (peak **5**), SME (peak **8**), genipin gentiobioside (peak **11**) and geniposide (peak **12**), one monocyclic monoterpenoid jasminoside B (peak **9**), one organic acid chlorogenic acid (peak **10**), one flavonoid rutin (peak **15**), and two crocins, crocin I (peak **20**) and crocin II (peak **25**). The mass spectrometry data of the components of peaks **1**, **6**, **7**, **13**, **14**, **17**–**19**, **21**–**24,** and **26**–**28** were the same as those reported in the previous literature.

For peak **16**, its quasimolecular ion was at a m/*z* of 597.1855 ([M-H]^−^) and a m/*z* of 633.1619 ([M+Cl]^−^), which was in accordance with the formula C_27_H_34_O_15_ based on its accurate mass. Li et al. also detected a component with the molecular formula C_27_H_34_O_15_ in GF by Q-TOF-MS and speculated that this component was penta-acetyl geniposide [10]. However, penta-acetyl geniposide is an artificial acetylated product from geniposide, which does not exist naturally in GF [35]. The [M-H]^−^ ion of peak **16** was selected as the precursor ion to generate MS/MS spectra, and fragment ions at m/*z* 597.1923, 391.1285, 229.0734, 223.0633, 205.0521, 185.0825, and 167.0717 were obtained. The ions at m/*z* 223.0633 (C_11_H_11_O_5_^−^) and 205.0521 (C_11_H_9_O_4_^−^) could be assigned as [sinapoyl-H]^−^ and [sinapoyl-H-H_2_O]^−^, suggesting the presence of a sinapoyl group in the molecule [36]. The ions at m/*z* 597.1923 (C_27_H_33_O_15_^−^) corresponding to [M-H]^−^ loss of a sinapoyl residue (C_11_H_10_O_4_^−^) yielded a predominant fragment ion at m/*z* 391.1285 (C_16_H_23_O_11_^−^), which was consistent with the precursor ion of shanzhiside. Fragment ions at m/*z* 229.0734 (C_10_H_13_O_6_^−^), 185.0825 (C_9_H_13_O_4_^−^), and 167.0717 (C_9_H_11_O_3_^−^) were produced by the ions at m/*z* 391.1285 with successive loss of a glucose unit (C_6_H_10_O_5_^−^), CO_2_, and H_2_O, respectively, which exhibited the same fragmentation pathway as shanzhiside. The abovementioned fragmentation pathways basically confirmed that the basic skeleton of the component of peak **16** was shanzhiside. Therefore, peak **16** was identified as a component of shanzhiside substituted by sinapoyl at 6′-O, and a natural compound with this kind of structure was also found in the compound database PubChem. Referring to the names of the components of peaks **17** and **24**, the component of peak **16** was temporarily named as 6′-trans-sinapoyl shanzhiside. To the best of our knowledge, this component was first detected in GF [1, 4–9].

### 3.4. Validation of Method for Quantitative Analysis

As given in Tables 3 and 4, the coefficient of determination values *R*^2^ was greater than 0.9995, all RSDs of the intraday precision, interday precision, stability, and repeatability were less than 5%, the average recovery rates were 96.37–102.65%, and the RSDs were 1.80–4.30%. The above results met the requirements of the standard drug quality analysis method in the Chinese Pharmacopoeia [38].

### 3.5. Wavelength Selection for Quantitative Analysis of 12 Components

**Figure 5:**
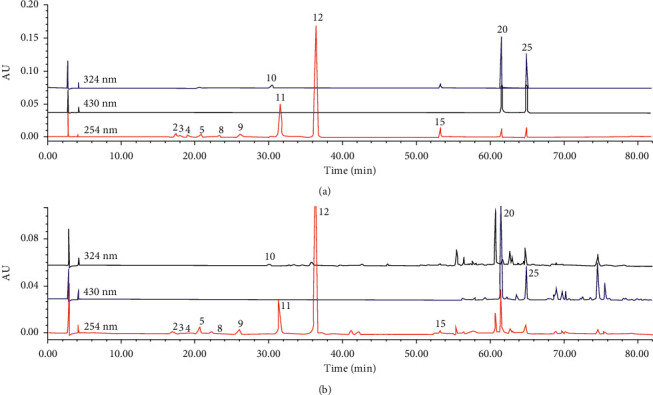
HPLC chromatograms of the mixed reference substances (a) and sample (b). The number of peaks is the same as in Table 2.

All 12 components could be detected at 254 nm, but the absorption of chlorogenic acid (peak 10) was stronger at 324 nm, and the absorptions of crocin I (peak 20) and crocin II (peak 25) were stronger at approximately 430 nm. Therefore, a wavelength of 324 nm was selected for the detection of chlorogenic acid, and a wavelength of 430 nm was selected for the detection of crocin I and crocin II. The chromatograms of the mixed reference substances and sample are shown in Figure 5.

### 3.6. Contents of 12 Representative Components in 40 Samples

As given in Table 5, there were certain differences in the contents of the 12 representative components among 40 samples, of which, the content of geniposide ranged from 37.917 to 72.216 mg/g, and the total content of the 7 iridoids ranged from 59.931 to 94.314 mg/g. Iridoids, especially geniposide, have both toxic and protective effects on the liver and kidney [16, 24–26, 39, 40]. It has been reported that the intragastric administration of 50 mg/kg/d bodyweight (human equivalent dose of 8 mg/kg/d bodyweight) geniposide in rats for 12 weeks can lead to liver and kidney damage [24]. According to this report, adults weighing 60 kg may suffer liver and kidney damage if they take 6 g or 10 g GF decoction pieces with a content of 80 mg/g or 48 mg/g every day for a long time. The recommended clinical dose of GF decoction pieces is 6–10 g/d in the Chinese Pharmacopoeia [2]. Table 5 provides that the geniposide content in most batches of GF decoction pieces exceeded 48 mg/g. Therefore, the content of representative components such as geniposide in GF decoction pieces should be measured before clinical use, and the dose of GF decoction pieces should be adjusted according to the content of these components to achieve a therapeutic effect and avoid adverse reactions.

## 4. Conclusion

In this study, QE of 40 batches of decoction pieces of GF produced by different manufacturers of herbal pieces is performed by qualitative analysis of the HPLC fingerprint and UFLC-triple-Q-TOF-MS/MS combined with quantitative analysis of multiple components, which we established previously for QE of traditional medicine. The results show that there are 28 common peaks in the HPLC fingerprints of 40 samples. The similarities between the sample chromatograms and reference chromatogram were higher. The components of these 28 common peaks are identified as 13 iridoids, 4 crocins, 7 monocyclic monoterpenoids, 3 organic acids, and 1 flavonoid. Of these, a total of 12 components, including the seven iridoids geniposide, shanzhiside, geniposidic acid, DAAMS, gardenoside, SME, and genipin gentiobioside, crocin I and crocin II, the monocyclic monoterpenoid jasminoside B, and the organic acid chlorogenic acid and the flavonoid rutin, were unambiguously identified by comparison with reference substances. There were certain differences in the contents of these 12 components among 40 samples; the geniposide content ranged from 37.917 to 67.039 mg/g, the total content of the 7 iridoids ranged from 37.917 to 67.039 mg/g, and the total content of 7 iridoids ranged from 59.931 to 87.843 mg/g. The content of representative components, such as geniposide, in GF decoction pieces should be measured before clinical use, and the dose of GF decoction pieces should be adjusted according to the content of these components to achieve a therapeutic effect and avoid adverse reactions.

## Figures and Tables

**Table 1:** Sample information and similarities.

Sample no.	Manufacturers	Batch no.	Origins of herb	Similarity
S1	Nantong Sanyue Herbal Pieces Co., Ltd.	171122	Jiangxi	0.999
S2	Nantong Sanyue Herbal Pieces Co., Ltd.	180402	Jiangxi	0.999
S3	Nantong Sanyue Herbal Pieces Co., Ltd.	200428	Jiangxi	0.993
S4	Nantong Sanyue Herbal Pieces Co., Ltd.	200616	Jiangxi	0.994
S5	Nantong Sanyue Herbal Pieces Co., Ltd.	200328	Jiangxi	0.994
S6	Nantong Sanyue Herbal Pieces Co., Ltd.	180131	Jiangxi	0.999
S7	Nantong Sanyue Herbal Pieces Co., Ltd.	180115	Jiangxi	0.996
S8	Suzhou Tianling Herbal Pieces Co., Ltd.	171222	Jiangxi	0.998
S9	Suzhou Tianling Herbal Pieces Co., Ltd.	171005010	Jiangxi	0.996
S10	Suzhou Tianling Herbal Pieces Co., Ltd.	151117010	Jiangxi	0.995
S11	Suzhou Tianling Herbal Pieces Co., Ltd.	16127010	Jiangxi	0.997
S12	Bozhou Baishixin Herbal Pieces Co., Ltd.	170601	Jiangxi	0.999
S13	Bozhou Qiaocheng Wanshixiang Herbal Pieces Co., Ltd.	180101	Jiangxi	0.995
S14	Anhui Xiehecheng Pharmaceutical Herbal Pieces Co., Ltd.	17110403	Jiangxi	0.999
S15	Hebei Renxin Pharmaceutical Co., Ltd.	22417008	Jiangxi	0.999
S16	Anhui Meiyu Herbal Pieces Co., Ltd.	111611027	Jiangxi	0.995
S17	Jiangxi Jiangzhong Herbal Pieces Co., Ltd.	171016	Jiangxi	0.998
S18	Jiangxi Jiangzhong Herbal Pieces Co., Ltd.	201222	Jiangxi	0.998
S19	Jiangxi Zhangshu Tianqitang Herbal Pieces Co., Ltd.	2010004	Jiangxi	0.989
S20	Anhui Puren Herbal Pieces Co., Ltd.	1709063	Jiangxi	0.998
S21	Anhui Puren Herbal Pieces Co., Ltd.	1711073	Jiangxi	0.997
S22	Anhui Fengyuan Tongling Herbal Pieces Co., Ltd.	15102002	Jiangxi	0.995
S23	Shanghai Kangqiao Herbal Pieces Co., Ltd.	180129	Jiangxi	0.998
S24	Suzhou Boyuan Pharmaceutical Co., Ltd.	150804–1	Jiangxi	0.997
S25	Anhui Huchuntang Herbal Pieces Co., Ltd.	150911	Jiangxi	0.997
S26	Bozhou Yonggang Herbal Pieces Co., Ltd.	171021	Jiangxi	0.998
S27	Bozhou Yonggang Herbal Pieces Co., Ltd.	160111201	Jiangxi	0.998
S28	Bozhou Yonggang Herbal Pieces Co., Ltd.	210602	Fujian	0.995
S29	Weiyuan Renze Pharmaceutical Co., Ltd.	200809	Fujian	0.996
S30	Fujian Mingyuan Pharmaceutical Co., Ltd.	201001	Fujian	0.992
S31	Jiangsu Longfengtang Herbal Pieces Co., Ltd.	20022831	Fujian	0.995
S32	Sichuan Tongshantang Herbal Pieces Co., Ltd.	190801	Sichuan	0.990
S33	Sichuan Zhongyong Pharmaceutical Co., Ltd.	201201	Sichuan	0.981
S34	Sichuan Gukang Pharmaceutical Co., Ltd.	201201	Sichuan	0.997
S35	Yancheng Herbal Pieces Co., Ltd.	2018011502	Hunan	0.998
S36	Hunan Nanguo Yaodu Herbal Pieces Co., Ltd.	170801	Hunan	0.997
S37	Nanning Shengyuan Herbal Pieces Co., Ltd.	210201	Guangxi	0.994
S38	Xuzhou Dapeng Herbal Pieces Co., Ltd.	200309	Guangxi	0.996
S39	Zhejiang Tongjuntang Herbal Pieces Co., Ltd.	151115	Zhejiang	0.998
S40	Tongling Hetian Herbal Pieces Co., Ltd.	20170413	Zhejiang	0.997

**Table 2:** Identification of the common peaks in the fingerprint by triple-Q-TOF-MS/MS.

Peak no.	*t*_R_ (min)	Formula	MS			MS/MS^c^	Identification	Types of compounds	Reference
			Measured	Theoretical	Error (ppm)				
1	15.330	C_16_H_22_O_10_	409.0910^a^	409.0907	0.7	193.0510, 373.1151, 167.0721, 149.0610, 89.0316, 211.0601, 161.0742, 179.0558, 123.0463, 409.0873, 143.0383	Gardenoside	Iridoids	[30, 36, 37]
2	17.542	C_16_H_24_O_11_	391.1249^b^	391.1246	0.8	391.1272, 185.0840, 167.0716, 229.0746, 119.0328, 89.0251, 211.0643, 149.0615, 179.0578, 123.0711	Shanzhiside^d^	Iridoids	
			427.1017^a^	427.1013	1.0	391.1273, 89.0261, 167.0721, 185.0825, 229.0728			

3	18.098	C_16_H_22_O_10_	409.0911^a^	409.0907	1.0	373.1163, 149.0619, 211.0617, 167.0718, 123.0466, 119.0355, 409.0875, 89.0252, 193.0534	Geniposidic acid^d^	Iridoids	
4	19.248	C_17_H_24_O_11_	439.1023^a^	439.1013	2.4	439.1033, 241.0684, 101.0240	DAAME^d^	Iridoids	
5	21.181	C_17_H_24_O_11_	439.1023^a^	439.1013	2.4	439.1088, 241.0733, 403.1295	Gardenoside^d^	Iridoids	
6	21.668	C_16_H_26_O_8_	345.1562^b^	345.1555	2.1	165.0915, 89.0249, 119.0343, 101.0267	Jasminoside D	Monocyclic monoterpenoids	[37]
			381.1333^a^	381.1322	3.0	165.0935, 345.1577, 89.0264, 179.0570, 119.0368, 101.0260, 121.1039			

7	22.668	C_16_H_24_O_9_	395.1114^a^	395.1114	-0.1	359.1353, 197.0822	Ixoroside	Iridoids	[37]
8	23.743	C_17_H_24_O_11_	439.1025^a^	439.1013	2.8	439.1056, 403.1332, 241.0704	SME^d^	Iridoids	
9	26.666	C_16_H_26_O_8_	381.1332^a^	381.1322	2.7	381.1332	Jasminoside B^d^	Monocyclic monoterpenoids	
10	30.424	C_16_H_18_O_9_	389.0648^a^	389.0645	0.5	191.0564, 353.0885	Chlorogenic acid^d^	Organic acids	
11	31.971	C_23_H_34_O_15_	585.1619^a^	585.1592	4.7	585.1644, 225.0778, 549.1867, 123.0462, 101.0257, 207.0671	Genipin gentiobioside^d^	Iridoids	
12	36.838	C_17_H_24_O_10_	423.1076^a^	423.1063	3.0	423.1095, 225.0760, 387.1288	Geniposide^d^	Iridoids	
13	42.163	C_16_H_26_O_7_	365.1387^a^	365.1373	4.0	365.1405	Jasminoside A	Monocyclic monoterpenoids	[37]
14	43.103	C_16_H_26_O_7_	365.1386^a^	365.1373	3.7	365.1385	Jasminoside E	Monocyclic monoterpenoids	[37]
15	53.572	C_27_H_30_O_16_	609.1482^b^	609.1461	3.4	609.1525, 301.0359	Rutin^d^	Flavonoids	
			645.1250^a^	645.1228	3.4	609.1501, 301.0366			

16	55.917	C_27_H_34_O_15_	597.1855^b^	597.1825	5.0	597.1923, 391.1285, 223.0633, 185.0825, 205.0521, 167.0717, 229.0734	6′-Trans-sinapoyl shanzhiside	Iridoids	
			633.1619^a^	633.1592	4.3	597.1881, 391.1260, 205.0511			

17	56.908	C_27_H_32_O_14_	615.1511^a^	615.1486	4.1	579.1770, 325.0927, 367.1043, 223.0612, 385.1151, 193.0505, 205.0506, 123.0451	6′-Trans-sinapoyl gardoside	Iridoids	[37]
18	59.556	C_25_H_24_O_12_	515.1215^b^	515.1195	3.9	353.0910, 191.0579, 515.1277, 179.0348, 173.0443, 161.0227, 135.04738, 155.0361	3,4-Dicaffeoylquinic acid	Organic acids	[30, 37]
19	61.039	C_32_H_40_O_17_	695.2208^b^	695.2193	2.2	695.2261, 469.1387, 163.0391, 145.0249, 123.0456, 367.1069, 225.0764, 663.2029,101.0252	6″-O-Trans-coumaroyl genipin gentiobioside	Iridoids	[36, 37]
20	61.814	C_44_H_64_O_24_	1011.3519^a^	1011.3482	3.7	1011.3585, 651.2726, 327.1621, 975.3809, 283.1722	Crocin I^d^	Crocins	
21	63.359	C_31_H_32_O_16_	659.1647^b^	659.1618	4.5	497.1325, 659.1672, 335.0767, 191.0555, 353.0892, 161.0458	3,4-Di-O-caffeoyl-5-O-(3-hydroxy-3-methylglutaroyl) quinic acid	Organic acids	[30, 37]
22	63.534	C_27_H_36_O_12_	587.1926^a^	587.1901	4.3	521.2075, 533.2077, 551.2697, 551.2697, 205.0517, 367.1050, 223.0621, 587.1956, 165.0928, 179.0727, 385.1160	6′-O-Trans-sinapoyl jasminoside L	Monocyclic monoterpenoids	[37]
23	64.290	C_21_H_34_O_11_	497.1810^a^	497.1795	3.0	497.1855, 461.2069, 167.1092, 329.0637, 293.0889	Jasminoside T	Monocyclic monoterpenoids	[37]
24	64.985	C_28_H_34_O_14_	593.1895^b^	593.1876	3.2	593.1943, 205.0518, 223.0649, 225.0781, 367.1057, 207.0645, 123.0466, 101.0236	6′-O-Trans-sinapoyl geniposide	Iridoids	[36, 37]
			629.1668^a^	629.1643	4.0	593.1936, 205.0511, 225.0773, 223.0627, 123.0457			
25	65.278	C_38_H_54_O_19_	849.2978^a^	849.2953	2.9	327.1615, 283.1723, 651.2701, 239.1815, 849.3028, 489.2166	Crocin II^d^	Crocins	
26	71.292	C_27_H_36_O_11_	571.1957^a^	571.1952	0.9	535.2258, 325.0947, 265.0740, 223.0627, 205.0521, 385.1184, 221.0848	6′-O-Trans-sinapoyl jasminoside A	Monocyclic monoterpenoids	[37]
27	75.160	C_44_H_64_O_24_	1011.3509^a^	1011.3482	2.7	1011.3556, 651.2711, 327.1611, 975.3767	13-cis-Crocin II	Crocins	[37]
28	75.958	C_32_H_44_O_14_	687.2437^a^	687.2425	1.7	327.1608, 651.2707, 283.1704, 687.2467, 239.1814, 323.0971, 179.0566	Crocin II	Crocins	[37]

^a^Quasi-molecular ion was [M+Cl]^−^. ^b^Quasimolecular ion was [M-H]^−^. ^c^Sequencing according to the abundance. ^d^Confirmed by comparison with reference substances.

**Table 3:** Results of the investigation of the linear relationship, LOD, and LOQ.

Reference substance	Regression equation	*R* ^2^	Linear range/ng	LOD/ng	LOQ/ng
Shanzhiside	*Y* = 410245X–1479	0.9998	25.78–1030	2.06	7.73
Geniposidic acid	*Y* = 664663X–107	0.9997	6–240	1.8	5.3
DAAME	*Y* = 604893X–2756	0.9999	8.806–352.24	1.76	7.04
Gardenoside	*Y* = 505562X–3934	0.9999	16.848–673.92	1.68	6.74
SME	*Y* = 615010X–1709	1.0000	7.196–287.84	2.16	7.2
Jasminoside B	*Y* = 989612X–7323	0.9995	12.1–484	2.42	6.05
Chlorogenic acid	*Y* = 2746452X–3099	0.9999	3.0768–123.072	0.92	3.69
Genipin gentiobioside	*Y* = 460657X–29122	0.9999	187.75–7510	3.76	13.14
Geniposide	*Y* = 746351X–82879	0.9999	380.6–15224	2.44	12.18
Rutin	*Y* = 1071542X–3095	0.9998	6.57–262.6	1.97	5.91
Crocin I	*Y* = 4453568X–29472	0.9999	20.25–810	0.61	2.03
Crocin II	*Y* = 4595808X–23116	0.9999	15–600	0.45	1.5

**Table 4:** Results of precision, stability, repeatability, and recovery tests (*n* = 6).

Components	Precision RSD (%)		Stability RSD (%)	Repeatability RSD (%)	Recovery	
	Intraday	Interday			Mean (%)	RSD (%)
Shanzhiside	0.87	0.98	4.96	0.97	96.58	1.80
Geniposidic acid	1.06	1.15	4.82	3.92	100.10	3.23
DAAME	0.80	0.91	3.63	1.35	98.11	2.86
Gardenoside	0.76	0.86	1.23	1.01	97.73	3.36
SME	0.85	0.95	4.84	2.05	98.17	1.89
Jasminoside B	1.25	1.37	2.26	2.07	98.46	2.74
Chlorogenic acid	1.16	1.07	3.54	2.88	102.65	3.61
Genipin gentiobioside	0.65	0.76	1.59	1.37	99.85	3.06
Geniposide	0.63	0.73	1.51	1.61	101.18	1.85
Rutin	0.50	0.59	4.69	2.85	96.37	4.30
Crocin I	0.48	0.56	1.33	2.57	99.05	3.76
Crocin II	0.49	0.56	1.36	2.20	96.51	3.59

**Table 5:** The contents of the 12 components in 40 samples (mg/g).

No.	Geniposide	Genipin gentiobioside	Gardenoside	Shanzhiside	DAAME	SME	Geniposidic acid	Total of iridoids^a^	Crocin I	Crocin II	Jasminoside B	Chlorogenic acid	Rutin
S1	56.157	17.254	3.324	1.616	0.279	0.240	0.288	79.159	7.733	0.879	1.184	0.175	0.452
S2	63.507	17.373	3.445	1.899	0.365	0.196	0.232	87.017	6.984	0.767	1.166	0.493	0.627
S3	47.306	8.479	3.230	0.767	0.214	0.174	0.137	60.308	7.640	1.156	0.794	0.437	1.043
S4	50.841	11.828	5.277	2.186	0.774	0.434	0.223	71.563	8.613	1.025	1.046	1.065	1.184
S5	49.480	10.326	5.043	2.159	0.821	0.452	0.246	68.526	8.122	0.905	0.956	1.386	1.315
S6	67.039	14.199	3.720	2.021	0.325	0.202	0.336	87.843	9.578	1.087	1.386	0.192	0.558
S7	57.153	20.159	3.293	1.405	0.622	0.297	0.412	83.341	6.061	1.251	1.023	0.187	0.595
S8	58.755	18.663	3.865	1.058	0.314	0.279	0.161	83.095	11.768	1.538	1.894	0.298	0.769
S9	56.628	20.945	3.687	1.737	0.277	0.183	0.244	83.701	5.586	0.909	0.947	0.275	0.536
S10	40.684	11.943	6.085	2.877	0.553	0.368	0.314	62.824	9.463	1.316	1.556	1.220	0.862
S11	49.570	12.767	3.688	1.487	0.479	0.348	0.189	68.526	8.798	1.226	1.259	0.152	0.492
S12	55.511	15.942	4.705	1.127	0.407	0.342	0.171	78.205	7.801	1.137	1.391	0.284	0.889
S13	45.203	8.848	6.438	2.488	0.802	0.256	1.499	65.534	9.038	1.578	0.949	1.900	0.478
S14	63.439	16.376	3.654	1.912	0.335	0.283	1.056	87.056	8.186	0.816	0.948	0.223	0.484
S15	57.478	14.724	2.873	1.854	0.335	0.259	0.166	77.690	10.132	1.200	1.377	0.137	0.514
S16	37.917	10.029	5.794	2.631	1.634	0.913	1.013	59.931	6.908	1.297	0.677	0.946	0.928
S17	50.662	16.733	2.693	0.936	0.565	0.404	0.132	72.124	5.735	0.777	0.562	0.566	0.491
S18	46.155	11.622	2.949	1.245	1.075	0.900	0.737	64.683	4.848	0.638	0.384	0.770	0.800
S19	46.483	9.444	5.048	2.334	0.447	0.327	0.170	64.253	9.612	1.667	1.411	0.667	0.936
S20	50.726	16.015	6.851	2.619	0.714	0.433	0.261	77.619	7.547	1.167	0.971	0.985	0.768
S21	47.560	16.348	6.315	2.257	0.872	0.551	0.250	74.153	7.438	1.055	1.086	0.734	0.665
S22	49.344	12.668	8.705	3.011	0.766	0.503	0.240	75.236	9.473	1.569	1.376	1.110	0.772
S23	61.648	15.332	3.138	1.005	0.541	0.200	0.169	82.033	11.266	1.334	1.352	0.456	0.475
S24	48.245	13.228	6.116	2.546	0.768	0.486	0.209	71.598	8.778	1.307	0.912	1.032	0.725
S25	51.362	10.919	5.128	1.502	0.527	0.368	0.256	70.062	6.948	0.952	0.790	0.476	0.742
S26	57.580	18.966	3.111	1.216	0.366	0.283	0.161	81.683	6.872	1.249	1.165	0.260	0.712
S27	50.456	13.112	3.050	1.777	0.455	0.264	0.130	69.244	10.195	1.564	1.214	0.175	0.531
S28	49.031	12.675	4.073	0.931	0.399	0.249	0.187	67.546	8.274	1.277	1.149	0.327	0.720
S29	51.922	10.231	3.632	1.167	0.469	0.348	0.277	68.045	7.475	1.024	0.920	0.618	1.007
S30	43.513	7.670	5.608	2.419	0.880	0.590	0.514	61.194	7.187	0.789	0.743	1.285	0.758
S31	54.676	9.316	4.095	1.180	0.598	0.378	0.347	70.590	6.558	1.202	1.238	0.624	1.601
S32	72.216	6.098	7.430	5.046	1.292	1.110	1.122	94.314	5.584	0.871	1.222	0.879	0.497
S33	56.122	5.352	7.029	2.854	0.739	0.561	0.474	73.131	12.837	2.978	2.015	0.584	0.334
S34	46.953	9.853	2.319	0.851	0.379	0.180	0.234	60.769	4.819	1.097	0.595	0.212	0.916
S35	56.624	15.340	0.623	1.180	5.596	1.505	0.401	81.270	6.752	1.142	0.122	0.227	0.800
S36	53.501	16.804	2.984	0.833	0.342	0.304	0.133	74.900	9.505	1.366	1.598	0.262	0.835
S37	47.872	9.927	3.013	0.885	0.564	0.353	0.141	62.754	7.241	1.305	1.148	0.379	1.002
S38	52.420	13.997	2.335	1.375	1.098	0.627	0.406	72.258	7.054	1.036	0.759	0.206	1.267
S39	50.352	13.880	5.154	0.288	0.453	0.351	0.197	70.675	6.587	0.882	1.036	1.001	0.575
S40	46.717	12.988	6.433	3.398	0.916	0.621	1.494	72.567	9.233	1.305	0.957	1.058	0.576
Average	52.470	13.209	4.399	1.802	0.734	0.428	0.383	73.425	8.006	1.191	1.082	0.607	0.756

^a^The sum of 7 iridoids.

## Data Availability

The data used to support the findings of this study are included within the article.

## References

[1] Chen L. P., Li M. X. (2020). Gardenia jasminoides ellis: ethnopharmacology, phytochemistry, and pharmacological and industrial applications of an important traditional Chinese medicine. *Journal of Ethnopharmacology*.

[2] Chinese Pharmacopoeia Commission (2020). *Chinese Pharmacopoeia*.

[3] Editorial Board of Japanese Pharmaceutical Bureau (2016). *Japanese Pharmacopoeia (XVII)*.

[4] Shu P. H., Yu M. Z., Zhu H. Q. (2021). Two new iridoid glycosides from Gardeniae Fructus. *Carbohydrate Research*.

[5] Cao Y. G., Ren Y. J., Liu Y. L. (2021). Iridoid glycosides and lignans from the fruits of Gardenia jasminoides Eills. *Phytochemistry*.

[6] Chen X., Cao Y. G., Ren Y. J. (2021). A new quinic acid derivative with *α*-glucosidase inhibitory activity from the fruit of Gardenia jasminoides. *Natural Product Research*.

[7] Li H. B., Ma J. F., Mei Y. D. (2022). Two new iridoid glycosides from the fruit of Gardenia jasminoides. *Natural Product Research*.

[8] Cao Y.-G., Zhang Y.-L., Zeng M.-N. (2020). Renoprotective mono- and triterpenoids from the fruit of gardenia jasminoides. *Journal of Natural Products*.

[9] Lu D., Zhang W., Jiang Y. (2019). Two new triterpenoids from Gardenia jasminoides fruits. *Natural Product Research*.

[10] Li W., Ren C., Fei C. (2021). Analysis of the chemical composition changes of Gardeniae Fructus before and after processing based on ultra‐high‐performance liquid chromatography quadrupole time‐of‐flight mass spectrometry. *Journal of Separation Science*.

[11] Zhou T., Zhao W., Fan G., Chai Y, Wu Y (2007). Isolation and purification of iridoid glycosides from Gardenia jasminoides Ellis by isocratic reversed-phase two-dimensional preparative high-performance liquid chromatography with column switch technology. *Journal of chromatography. B, Analytical technologies in the biomedical and life sciences*.

[12] Wang Y., Chen Y., Deng L. (2015). Systematic separation and purification of iridoid glycosides and crocetin derivatives from Gardenia jasminoides Ellis by high-speed counter-current chromatography. *Phytochemical Analysis*.

[13] Song Y. N., Wang Y., Zheng Y. H. (2021). Crocins: a comprehensive review of structural characteristics, pharmacokinetics and therapeutic effects. *Fitoterapia*.

[14] Chen Q. C., Youn U., Min B. S., Bae K. (2008). Pyronane monoterpenoids from the fruit of gardenia jasminoides. *Journal of Natural Products*.

[15] Ouyang E., Li X., Zhang C. (2011). Simultaneous determination of geniposide, chlorogenic acid, crocin1, and rutin in crude and processed Fructus Gardeniae extracts by high performance liquid chromatography. *Pharmacognosy Magazine*.

[16] Dong R., Tian Q., Shi Y. (2021). An integrated strategy for rapid discovery and identification of quality markers in gardenia fructus using an omics discrimination-grey correlation-biological verification method. *Frontiers in Pharmacology*.

[17] Chen P., Chen Y., Wang Y. (2016). Comparative evaluation of hepatoprotective activities of geniposide, crocins and crocetin by CCl4-induced liver injury in mice. *Biomolecules & Therapeutics*.

[18] Saravanakumar K., Park S. J., Sathiyaseelan A. (2021). Metabolite profiling of methanolic extract of gardenia jaminoides by LC-MS/MS and GC-MS and its anti-diabetic, and anti-oxidant activities. *Pharmaceuticals*.

[19] Wang L., Yang C., Song F., Liu Z, Liu S (2020). Therapeutic effectiveness of gardenia jasminoides on type 2 diabetic rats: mass spectrometry-based metabolomics approach. *Journal of Agricultural and Food Chemistry*.

[20] Xia B. M., Huang X. Y., Sun G. D., Tao W. W. (2021). Iridoids from Gardeniae fructus ameliorates depression by enhancing synaptic plasticity via AMPA receptor-mTOR signaling. *Journal of Ethnopharmacology*.

[21] Guo S., Bao L., Li C., Sun J, Zhao R, Cui X (2020). Antiviral activity of iridoid glycosides extracted from Fructus Gardeniae against influenza A virus by PACT-dependent suppression of viral RNA replication. *Scientific Reports*.

[22] Shi Y. P., Zhang Y. G., Li H. N. (2020). Discovery and identification of antithrombotic chemical markers in Gardenia Fructus by herbal metabolomics and zebrafish model. *Journal of Ethnopharmacology*.

[23] Ni Y., Li L., Zhang W. Y. (2020). Discovery and LC-MS characterization of new crocins in gardeniae fructus and their neuroprotective potential. *Journal of Agricultural and Food Chemistry*.

[24] Li C. N., Gao X., Gao X. C. (2021). Effects of medicine food Fructus Gardeniae on liver and kidney functions after oral administration to rats for 12 weeks. *Journal of Food Biochemistry*.

[25] Li C., Lan M., Lv J. (2019). Screening of the hepatotoxic components in fructus gardeniae and their effects on rat liver BRL-3A cells. *Molecules*.

[26] Tian J., Yi Y., Zhao Y. (2018). Oral chronic toxicity study of geniposide in rats. *Journal of Ethnopharmacology*.

[27] Liu H., Chen Y.-F., Li F., Zhang H.-Y. (2013). Fructus Gardenia (Gardenia jasminoides J. Ellis) phytochemistry, pharmacology of cardiovascular, and safety with the perspective of new drugs development. *Journal of Asian Natural Products Research*.

[28] Yin F., Wu X., Li L. (2015). Quality control of gardeniae fructus by HPLC-PDA fingerprint coupled with chemometric methods. *Journal of Chromatographic Science*.

[29] Wu X., Zhou Y., Yin F. (2014). Quality control and producing areas differentiation of Gardeniae Fructus for eight bioactive constituents by HPLC-DAD-ESI/MS. *Phytomedicine*.

[30] Han Y., Wen J., Zhou T., Fan G. (2015). Chemical fingerprinting of Gardenia jasminoides Ellis by HPLC-DAD-ESI-MS combined with chemometrics methods. *Food Chemistry*.

[31] Coran S. A., Mulas S., Vasconi A. (2014). Profiling of components and validated determination of iridoids in gardenia jasminoides ellis fruit by a high-performance-thin-layer- chromatography/mass spectrometry approach. *Journal of Chromatography A*.

[32] Lee E. J., Hong J. K., Whang W. K. (2014). Simultaneous determination of bioactive marker compounds from gardeniae fructus by high performance liquid chromatography. *Archives of Pharmacal Research*.

[33] Dai Y., Dou Z. H., Zhou R. R. (2021). Quality evaluation of Artemisia capillaris Thunb. based on qualitative analysis of the HPLC fingerprint and UFLC-Q-TOF-MS/MS combined with quantitative analysis of multi-components. *Journal of Analytical Methods in Chemistry*.

[34] SFDA (State Food and Drug Administration of China) (2000). *Technical Requirements for the Development of Fingerprints of TCM Injections*.

[35] Peng C., Huang C., Wang C. (2005). The anti-tumor effect and mechanisms of action of penta-acetyl geniposide. *Current Cancer Drug Targets*.

[36] Fu Z., Xue R., Li Z. (2014). Fragmentation patterns study of iridoid glycosides in Fructus Gardeniae by HPLC-Q/TOF-MS/MS. *Biomedical Chromatography*.

[37] Wang L., Liu S., Zhang X., Xing J., Liu Z., Song F. (2016). A strategy for identification and structural characterization of compounds from gardenia jasminoides by integrating macroporous resin column chromatography and liquid chromatography-tandem mass spectrometry combined with ion-mobility spectrometry. *Journal of Chromatography A*.

[38] China Medical Science Press (2020). *Pharmacopoeia of China, Part 4*.

[39] Li F., Chen Y., Li Y., Huang M, Zhao W (2020). Geniposide alleviates diabetic nephropathy of mice through AMPK/SIRT1/NF-*κ*B pathway. *European Journal of Pharmacology*.

[40] Mahgoub E., Kumaraswamy S. M., Kader K. H. (2017). Genipin attenuates cisplatin-induced nephrotoxicity by counteracting oxidative stress, inflammation, and apoptosis. *Biomedicine & Pharmacotherapy*.

